# CONSIDERATIONS OF AGE-FRIENDLY 4M PRINCIPLES IN DEPRESCRIBING INTERVENTIONS: A SCOPING REVIEW

**DOI:** 10.1093/geroni/igac059.2028

**Published:** 2022-12-20

**Authors:** Jinjiao Wang, Jenny Shen, Yeates Conwell, Kobi Nathan, Fang Yu, Erika Ramsdale, Donna Fick, Sandra Simmons

**Affiliations:** University of Rochester School of Nursing, Rochester, New York, United States; University of Rochester Medical Center, Rochester, New York, United States; University of Rochester Medical Center, Rochester, New York, United States; St. John Fisher College, Rochester, New York, United States; Arizona State University, Phoenix, Arizona, United States; University of Rochester Medical Center, Rochester, New York, United States; Penn State University, University Park, Pennsylvania, United States; Vanderbilt University Medical Center, Nashville, Tennessee, United States

## Abstract

The 4Ms – Medication, Mentation, Mobility, and What Matters - represent the key components in the Age-Friendly Health Systems initiative and provide a conceptual framework for research in the older adult population. Over 40% of older adults experience polypharmacy, which can be addressed by deprescribing unnecessary medications. This review aimed to assess the degree to which the 4Ms were considered in intervention design, sample selection, and outcome assessment in deprescribing trials by keyword search in six databases and snowballing. Thirty-seven of the 564 trials identified met the review eligibility criteria. Imbalanced consideration of 4Ms in the deprescribing trials was observed. Intervention design: “Medication” was considered in all trials; “Mentation” was considered in 8 trials; “Mobility” (n=2) and “What Matters” (n=6) was less often considered. By targeting providers, most of the trials lacked consideration of patient-centeredness - aligning what matters most to older adults and their families with deprescribing decision making and implementation. Sample selection: “Medication” was considered in 15 trials (e.g., samples including patients taking ≥ 5 medications or specific types of medications), whereas “Mentation” (n=6), “Mobility” (n=6) and “What Matters” (n=0) were less often considered. Outcome assessment: “Medication” was the most commonly assessed outcomes (n=33), followed by “Mobility” (n=13) and “Mentation” (n=10) outcomes, with no study examining “What Matters” outcomes. 4Ms were not purposefully considered in the intervention design, sample selection, and outcome measurement of existing deprescribing trials. Future deprescribing trials need a more balanced and complete consideration of the 4Ms in the trial design and implementation.

